# *The Organon* and Materica Medica Club of the Bay Cities of California

**Published:** 1894-10

**Authors:** 


### *THE ORGANON* AND MATERIA MEDICA CLUB OF THE BAY CITIES OF CALIFORNIA.

This is the name of a new organization started for mutual improvement of its members, by the discussion of medical subjects and the relating of experiences in the use of homœopathic methods of treatment.

The society was organized after the issuing by Dr. J. M. Selfridge of the following letter of invitation:

OAKLAND, July 20th, 1894.

DEAR DOCTOR:—The formation of a club, having as its object the advancement of pure Homœopathy, by the study of *The Organon* and kindred subjects, has been talked of, now and again, for years by some of the physicians in and out of San Francisco, but more especially so during the last few months, until now it has been suggested that the undersigned open correspondence with a certain few, for the purpose of ascertaining their views on this subject. It has not been thought desirable to have the club consist of more than eight or twelve members, so that, without inconvenience, the club can meet at the offices of the members at such times as they may elect.

It is thought that the test for membership should be an honest belief in the doctrines of Hahnemann, and in *practice* the use of the *single* remedy, while the size of the dose should be left to the judgment of the practitioner.

Is it your wish to become an active member of such a club? Kindly answer at your earliest convenience, and oblige,

Very respectfully and fraternally,

P. O. Box 37. (Signed) J. M. SELFRIDGE.

In relation to this club the Secretary, Dr. W. E. Ledyard, writes:

223 Post Street, San Francisco, Sept. 22d, 1894.

Editor of The Homœopathic Physician:—In accordance with your request, I gratefully enclose a record of the transactions of "*The Organon* and Materia Medica Club of the Bay Cities of California," to be published in The Homœopathic Physician.

In these few lines I take pleasure in stating that I feel confident that the above "club" will supply a want long felt on this coast.

Every member of the club is full of zeal and enthusiasm for the good cause, and looks forward to the time when Quinine, Morphine, "*et hoc genus omne*," will be a thing of the past, to be remembered only as a horrible nightmare; to the time when ruddy health will bear testimony to the truth as exemplified in "*similia similibus curantur*."

Believe me to be, as ever, Most fraternally yours,

W. E. Ledyard.

---

